# Identification of ZIC2 as a Potential Biomarker Linked with the Clinical Progression and Immune Infiltration of Oral Cancer: A Multicenter Study

**DOI:** 10.1155/2024/3256694

**Published:** 2024-01-24

**Authors:** Li Gao, Shi-Bai Yan, Fang-Cheng Jiang, Zhi-Guang Huang, Dong-Ming Li, Yu-Lu Tang, Jia-Yuan Luo, Gang Chen, Dan-Ming Wei

**Affiliations:** Department of Pathology, The First Affiliated Hospital of Guangxi Medical University, No. 6 Shuangyong Rd, Nanning, Guangxi Zhuang Autonomous Region 530021, China

## Abstract

**Aim:**

To investigate the specific expression profile, clinicopathological significance and mechanism of Zic family member 2 (ZIC2) in oral cancer were unclear. *Patients and Methods*. We explored the expression pattern and clinicopathological significance of ZIC2 in oral cancer through performing in-house tissue microarray and integrated analysis global RNA-seq and microarrays containing large samples. The molecular basis of ZIC2 in oral cancer was further investigated in the aspects of transcription network and immune correlations. We also performed in vitro experiments and calculated drug sensitivity of oral cancer with different ZIC2 expression levels in response to hundreds of compounds.

**Results:**

All data unanimously proved the significant overexpression of ZIC2 in oral cancer. The upregulation of ZIC2 was remarkably associated with the malignant clinical progression of oral cancer. ZIC2 was predicted to be targeted by miRNAs such as miR-3140, miR-4999, and miR-1322. The infiltration level of CD8+ T and central memory cells was positively related to the overexpression of ZIC2. Oral cancer patients with higher ZIC2 expression showed higher drug sensitivity to two compounds including AZD8186 and ERK_2240.

**Conclusions:**

We demonstrated the upregulation of ZIC2 in oral cancer and its promoting effect on the clinical advancement of oral cancer. The potential clinical value of ZIC2 in oral cancer deserves attention.

## 1. Introduction

Oral cancer ranked as the sixth most common cancer worldwide [1]. In China, new incidences of oral cancer are about 45200 in 2019, and it is increasing every year [2]. A large number of epidemiological studies have shown that unclean mouth, malnutrition, ulcer, drinking, and smoking are the main causes of oral cancer [3]. Surgery was adopted as a main treatment for oral cancer patients [4]. For oral cancer patients in the middle and late stage, the regimen of surgery combined with radiotherapy or chemotherapy was applied [4]. Although the curative effect of surgical treatment is relatively good in oral cancer patients at early stage, early oral cancer is hard to be diagnosed, which reduces the survival rate of patients with oral cancer [5, 6]. The 5-year survival probability of patients with advanced oral cancer still has not reach 63% [7]. Therefore, it has become a hot issue to clarify the pathogenesis of oral cancer and develop novel targeted treatments.

Zic family member 2 (ZIC2) is a member of the ZIC family of proteins with highly conserved cysteine 2/histidine 2 motifs, functioning as transcriptional regulators with crucial roles in diverse biological processes and cellular functions including embryo development, cell morphogenesis, skeletal patterning, and neurogenesis [8]. There is mounting evidence of the oncogenic influence of ZIC2 expression in human cancers, and high expression of ZIC2 has been found in nasopharyngeal cancer, breast cancer, and prostate cancer [9–11]. Overexpression of ZIC2 was intimately associated with the invasion, metastasis, and self-renewal of cancer cells [12, 13]. However, the specific expression profile, clinicopathological significance, and mechanism of ZIC2 in oral cancer were unclear.

In the current study, the expression pattern and clinicopathological significance of ZIC2 in oral cancer were clearly characterized through integrated analysis of in-house tissue microarray, global RNA-seq, and microarrays containing large samples. The molecular basis of ZIC2 in oral cancer was further investigated in the aspects of transcription network and immune correlations. We also performed in vitro experiments and calculated drug sensitivity of oral cancer with different ZIC2 expression levels in response to hundreds of compounds.

## 2. Materials and Methods

### 2.1. In-House Tissue Microarray

A total of 159 surgical resected oral cancer tissues and 48 noncancer oral tissues (including 14 cases of normal oral tissues, four cases of inflammation, eight cases of noncancerous squamous epithelium, 12 cases of pleomorphic adenoma, five cases of schwannoma, three cases of neurofibroma, and two cases of hemangioma (date: January 2018 to June 2019)) were acquired from Guilin Fanpu Biotech of Guangxi, China (Supplementary Table 1). All patients involved in the tissue microarray experiments signed informed consents. The ethics committee of Guilin Fanpu Biotech approved the current study.

Detailed methods of performing the immunohistochemistry (IHC) experiments for each sample were recorded in previous studies [14, 15]. Two experienced pathologists reviewed and evaluated the immunostaining scores of all slides independently. All the cells in a field were counted for evaluation of immunostaining, and five fields were counted for each slide. The IHC score was expressed as the product of the scores of positive staining area and the scores for staining intensity (0, negative; 1, weak; 2, medium; and 3, strong). The percentage of positive staining area ranging from <5%, 5-25%, 26-50%, 51-75%, to >75% corresponded to the scores of 0-4. Scores of staining intensity ranging from 0 to 3 represented negative, weak, medium, and strong immunostaining, respectively. Low and high ZIC2 protein expressions were judged according to the cutoff IHC score of 4 (IHC score < 4: low expression; IHC score ≥ 4: high expression). The positive and negative controls used for evaluation of IHC results are displayed in Supplementary Figure 1.

### 2.2. Curation of Public Microarrays and RNA-Seq Datasets

We downloaded the gene expression matrix (in the format of fragments per kilobase per million (FPKM)) and the matched clinical data of oral cancer patients from GDC data portal (https://portal.gdc.cancer.gov/repository) of The Cancer Genome Atlas (TCGA) database. The FPKM gene expression matrix was transformed to transcripts per million (TPM) gene expression matrix and standardized with the function of log2 (TPM + 0.001). Microarrays in ArrayExpress (https://www.ebi.ac.uk/arrayexpress/) or Gene Expression Omnibus (GEO) (https://www.ncbi.nlm.nih.gov/gds/?term=) databases published before February 24, 2022, were included for differential expression analysis if they contained ZIC2 expression value in at least three human oral cancer and noncancer oral samples. We set the number of three as the cutoff value because at least three samples in each group were required for valid differential expression analysis.

### 2.3. Integration of Tissue Microarray, External Microarrays, and RNA-Seq Datasets for Comprehensive Analysis of ZIC2 Expression

ZIC2 expression value in oral cancer and noncancer oral samples from all included microarrays was sorted and compiled according to the methods described before [16, 17]. The overall differential expression of ZIC2 between oral cancer and noncancer oral tissues of in-house tissue microarray and public microarrays or RNA-seq datasets was analyzed through calculation of standard mean difference (SMD) with 95% confidence interval (CI), which was conducted by the meta package in R software v.3.6.1. The distinguishing capacity of ZIC2 expression for oral cancer samples was evaluated with the summarized receiver operating characteristic (SROC) curves drawn by Stata v.14.0 [18].

### 2.4. The Prognostic Significance of ZIC2 in Oral Cancer

Microarrays and RNA-seq datasets with overall survival information of oral cancer patients plus the matched expression value of ZIC2 were retrieved from TCGA, ArrayExpress, and GEO databases. The prognostic data extracted from the included datasets was sorted for further univariate Cox regression analysis through R packages of ggplot2, survminer, and survival. Oral cancer patients were separated based on median normalized ZIC2 expression value in oral cancer tissues for the Kaplan-Meier survival analysis. All hazard ratio (HR) values derived from univariate Cox regression analysis were pooled for calculation of overall HR value and displayed as forest plot by meta package in R software.

### 2.5. The Subcellular Localization and Coexpressed Genes of ZIC2

The distribution of ZIC2 in different regions of cells and coexpressed genes of ZIC2 was figured out through referring to GeneCards and STRING databases.

### 2.6. The Upstream Regulators and Downstream Transcriptional Binding Sites of ZIC2

We predicted miRNAs that potentially regulate ZIC2 expression via TransmiR database. Considering the attribute of ZIC2 as a transcription factor, we also explored the possible binding sites between ZIC2 and its target genes according to the FASTA promoter sequence of ZIC2. The frequency matrix of four bases in the predicted binding sites was generated by JASPAR database.

### 2.7. The Associations between ZIC2 Expression and Immune Infiltration of Oral Cancer

Firstly, the abundance of various tumor-infiltrating immune cells of oral cancer samples from TCGA database was calculated through ImmunecellAI based on the normalized TPM gene expression matrix. The correlation between ZIC2 expression and the 24 immune cells in oral cancer was analyzed with the Pearson correlation test and visualized using corrplot package of R software. Furthermore, the association between copy number of ZIC2 and B cells, macrophages, CD8+ T cells, neutrophils, CD4+ T cells, or dendritic cells in the head and neck cancer was investigated utilizing TIMER database.

### 2.8. Drug Sensitivity Prediction for Oral Cancer Patients with Different Levels of ZIC2 Expression

To select effective drugs for oral cancer patients bearing high ZIC2 expression, we predicted the sensitivity of oral cancer samples in TCGA database to a total of 179 drugs with oncoPredict package of R software. Training set was acquired from the expression matrix and drug treatment information in Genomics of Drug Sensitivity in Cancer (GDSC) project v.2. For the testing set, the median expression value of ZIC2 in oral cancer patients from TCGA database served as grouping threshold. The Wilcox test was applied for comparing the drug sensitivity between the low expression and the high expression group. Drugs showing high affinity to oral cancer patients with high ZIC2 expression were screened (*P* < 0.05 and log2FC), and the corresponding box plots of drug sensitivity was exhibited with ggplot package in R software.

### 2.9. Functional Annotations for Genes Differentially Expressed in High and Low ZIC2 Expression Groups

Differential expression analysis was conducted on expression matrix of 341 oral cancer samples in RNA-seq dataset, which was divided by the mean expression value of ZIC2. The log2 normalized TPM expression matrix was treated by limma package, and genes with significant differential expression (|log2FC| > 0.1 and adj. *P* value < 0.05) between high and low ZIC2 expression groups were selected for further gene set enrichment analysis (GSEA). The enrichment of these genes in biological functions and KEGG pathways was annotated by clusterProfiler package of R software v.4.1.0. Terms with absolute normalized enrichment score (NES) of >1 and *P* value of < 0.05 were significant.

### 2.10. Culture of Cal-27 Cells

The Cal-27 cell line is seeded in a 100 mm culture dish with DMEM medium containing 10% fetal bovine serum (FBS), 100 IU/mL penicillin, and 100 IU/mL streptomycin and cultured in an incubator in a humid environment of 5% CO_2_.

### 2.11. Transfection of ZIC2 siRNA into Cal-27 Cells

Cal-27 cells were routinely cultured in DMEM medium containing 10% fetal bovine serum and placed in a 37°C constant temperature incubator with 5%CO_2_. When cell confluence reaches 70%, 40 nmol/L ZIC2 siRNA and negative control (NC) siRNA were transfected into Cal-27 cells according to the instructions of the RNATransMate reagent (Sangon Biotechnology Co., Ltd., Shanghai). The sequences of ZIC2 siRNA were 5′-GCAACUGAGCAAUCCCAAGAATT-3′ (sense) and 5′-UUCUUGGGAUUGCUCAGUUGCTT-3′ (antisense) (Sangon Biotechnology Co., Ltd., Shanghai). The sequences of NC siRNA were 5′-UUC UCCGAACGUGUCACGUTT-3′ (sense) and 5′-ACGUGACACGUUCGGAGAATT-3′ (antisense) (Sangon Biotechnology Co., Ltd., Shanghai). After 48 hours of transfection, Cal-27 cells were digested with trypsin containing 0.25%EDTA, and the total RNA of all cells was extracted using Tiangen RNAsimple total RNA kit (DP419, TIANGEN Biotech CO., Ltd., Beijing, China). The concentration of RNA was measured using NanoDrop 2000. According to the instructions of PrimeScript™ RT reagent Kit with gDNA Eraser (Perfect Real Time) (RR047A, Takara Bio), cDNA was synthesized with 1 *μ*g of RNA. With GAPDH as the internal reference, the mRNA expression of ZIC2 was detected using SYBR Green I dye on the Bio-Rad CFX96 system. Gene expression in each sample was calculated using the 2^(-*ΔΔ*)^ CT method. The forward and reverse sequences of ZIC2 primers are 5′-CGGGCTGTGGCAAAGTCTTCG-3′ and 5′-CTTCTTCCTGTCGCTGCTGTTGG-3′ (Sangon Biotechnology Co., Ltd., Shanghai). The forward and reverse sequences of GAPDH primers are 5′-GCACCGTCAAGGCTGAGAAC-3′ and 5′-TGGTGAAGACGCCAGTGGA-3′ (Takara Bio). If the mRNA expression of ZIC2 in cells of the ZIC2 siRNA group decreases by more than 70% compared with the NC siRNA group, the knockdown is regarded successful.

### 2.12. CCK8 Assay

After 24 hours of transfection with ZIC2 siRNA or NC siRNA, Cal-27 cells were seeded into 96 well plates at a density of 2 × 10^3^ cells per well. Six parallel wells were set for each group, and the plates were incubated at 37°C for 24, 48, 72, 96, and 120 hours. Subsequently, 10 *μ*L CCK-8 reagent (CK04, DOJINDO) was added to each well. The absorbance value at a wavelength of 450 nm (*A*_450 nm_) was determined through a microplate reader after two hours of incubation at 37°C.

### 2.13. Scratch Wound Assay

Cal-27 cells transfected with ZIC2 or NC siRNA for 48 hours were inoculated into a 6-well culture plate at a density of 1 × 10^5^ cells/well. The scratch in each well was made with 100 *μ*L pipette tips by vertical force when the cells covered 90% area of each well. Then, the cells were washed with PBS for three times, and serum-free DMEM medium was added to all wells. Cell migration at 0 h and 24 h was observed by taking photos under a microscope at a magnification of ×200.

### 2.14. Transwell Invasion Assay

The Matrigel gel was mixed with serum-free DMEM medium at the ratio of 1 : 5, and 80 *μ*L diluted Matrigel was added to the Transwell chamber on the Transwell plate. The 24-well Transwell plate was placed in a 37°C incubator for several hours until solidification. Then, serum-free suspension of Cal-27 cells transfected with ZIC2 siRNA or NC siRNA (5 × 10^5^/mL) in a volume of 200 *μ*L was added to the upper chamber. DMEM medium containing 10% FBS was added to the lower chamber of each well (600 *μ*L). Cells in the 24-well plate were cultured for 48 h in a 37°C incubator. Subsequently, we took out the Transwell chamber, washed it twice with PBS, wiped off the cells on the upper surface with a cotton swab, fixed the cells with anhydrous methanol for 30 minutes, and stained the cells with 0.1% crystal violet staining solution for 24 hours. After washing with PBS and air-drying, photos of cells passing through the basement membrane were captured under a fluorescent-inverted microscope at a magnification of ×200.

### 2.15. Statistical Analysis

The distribution of ZIC2 in different clinicopathological groups of oral cancer patients from in-house tissue microarray and TCGA database was analyzed through independent sample's *t* test, Mann–Whitney test, and analysis of variance in SPSS 22.0. Expression value of ZIC2 was summarized in mean ± standard deviation. *P* < 0.05 means statistical significance.

## 3. Results

### 3.1. The Expression Landscape of ZIC2 in Oral Cancer

**Figure 1:**
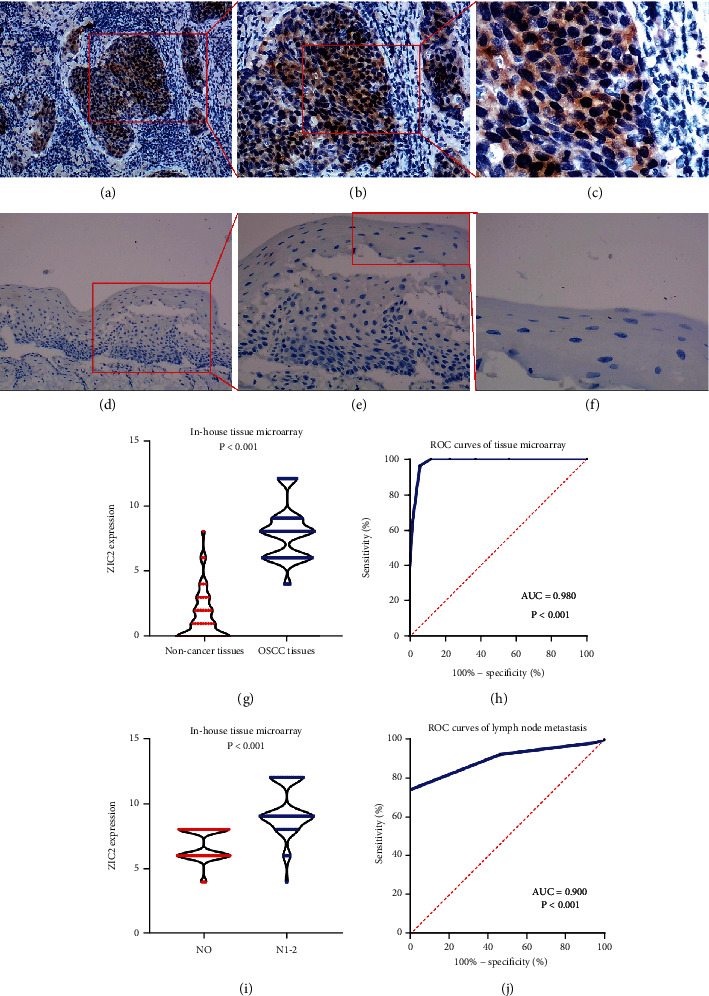
Expression pattern of ZIC2 in oral cancer samples from in-house tissue microarray: (a) immunohistochemistry pictures representing high protein level of ZIC2 in oral cancer tissues (×100); (b) immunohistochemistry pictures representing high protein level of ZIC2 in oral cancer tissues (×200); (c) immunohistochemistry pictures representing high protein level of ZIC2 in oral cancer tissues (×400); (d) immunohistochemistry pictures representing low protein level of ZIC2 in noncancer oral tissues (×100); (e) immunohistochemistry pictures representing low protein level of ZIC2 in noncancer oral tissues (×200); (f) immunohistochemistry pictures representing low protein level of ZIC2 in noncancer oral tissues (×400); (g) box plot showing the comparison of ZIC2 expression between oral and noncancer oral tissues; (h) ROC curves of the discerning ability of ZIC2 expression for oral cancer tissues; (i) box plot showing the comparison of ZIC2 expression between oral cancer patients with or without lymph node metastasis; (j) ROC curves of the discerning ability of ZIC2 expression for oral cancer patients with lymph node metastasis.

**Figure 2:**
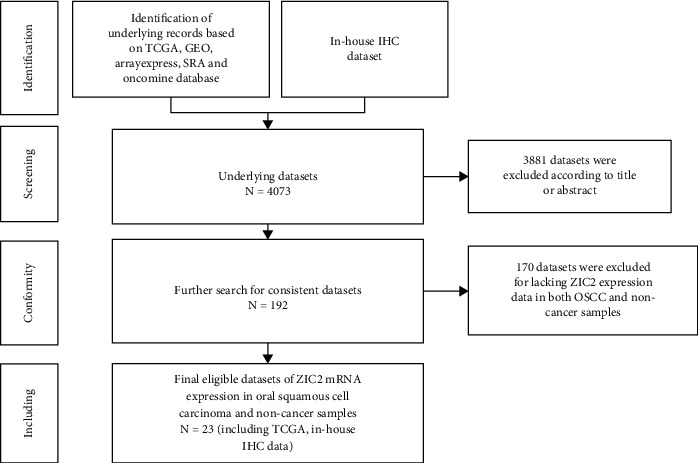
Flowchart of the selection process of external RNA-seq dataset and microarrays.

**Figure 3:**
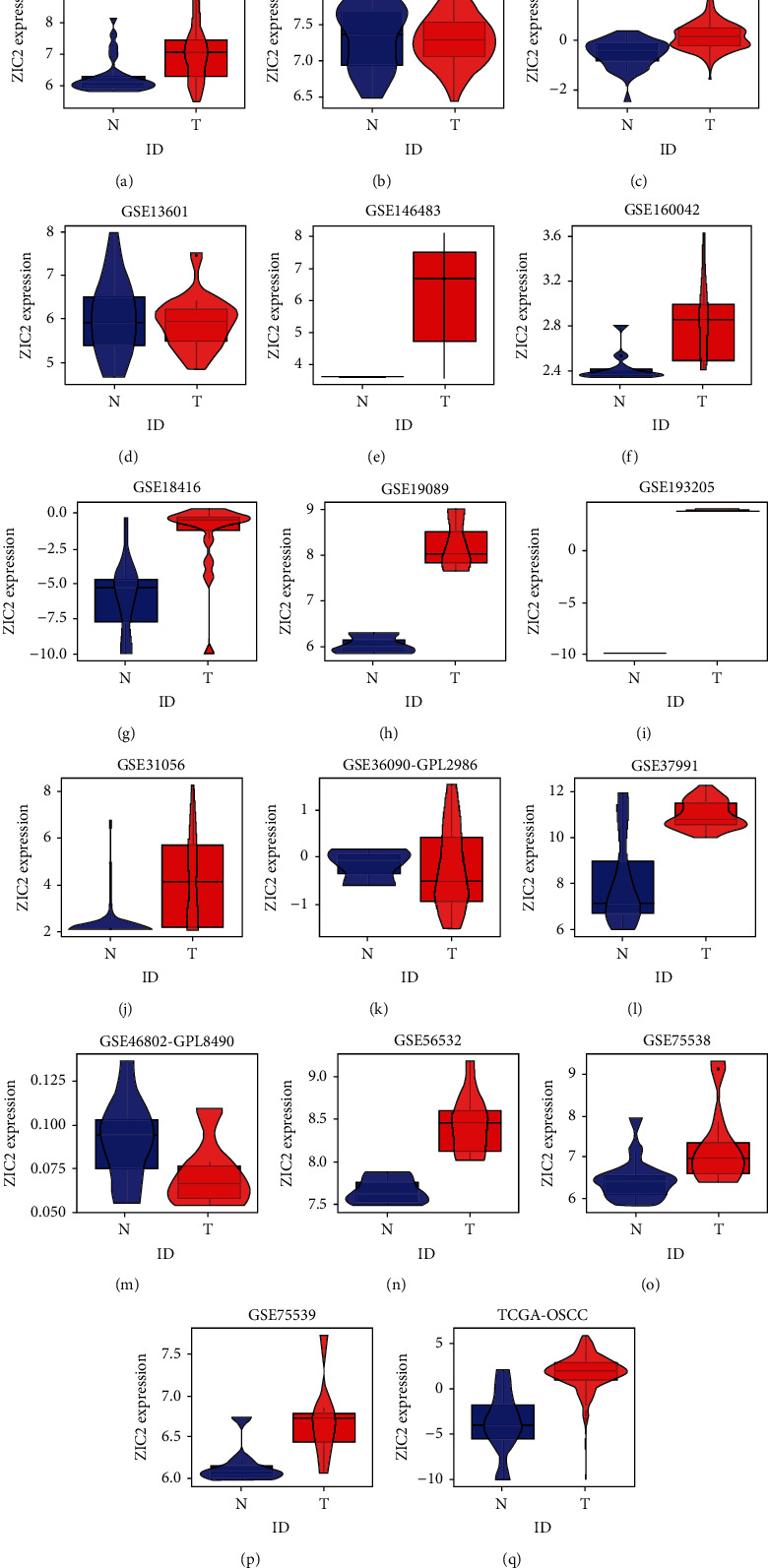
Differential expression of ZIC2 in oral cancer and noncancer oral samples from external RNA-seq dataset and microarrays. N: noncancer samples; T: tumor samples.

**Figure 4:**
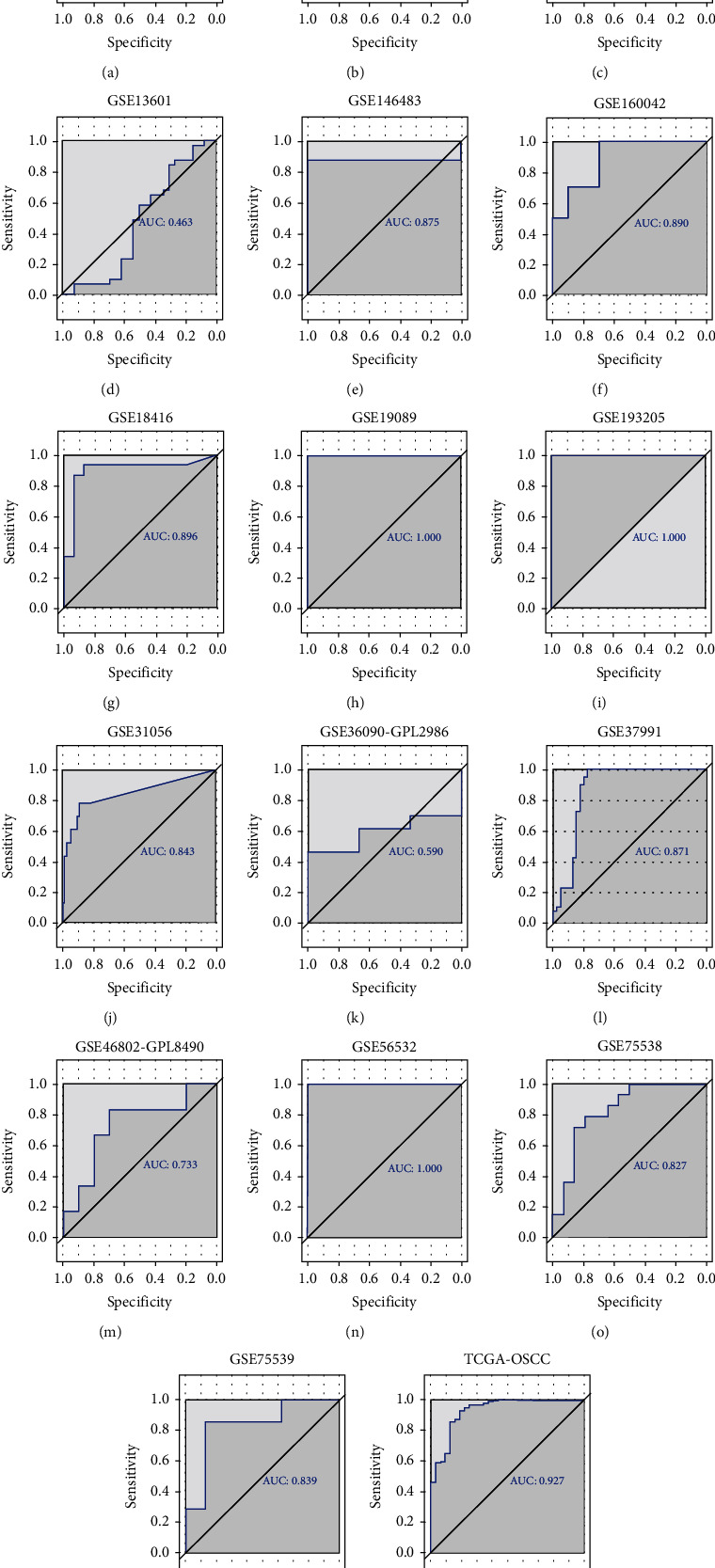
ROC curves of the discerning ability of ZIC2 expression for oral cancer patients from external RNA-seq dataset and microarrays. AUC: area under curve.

**Figure 5:**
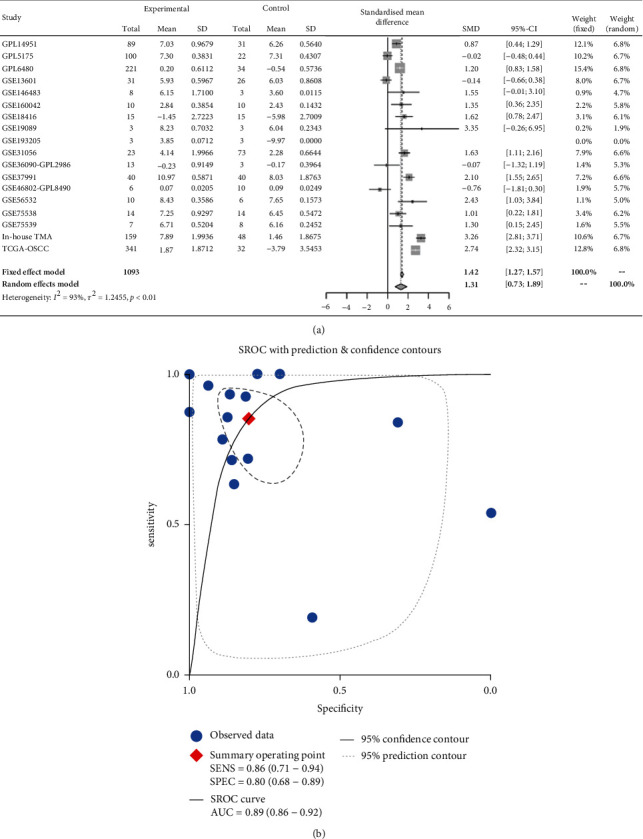
The overall upregulation of ZIC2 in oral cancer samples and the distinguishing ability of ZIC2 overexpression for oral cancer tissues: (a) forest plot of standard mean difference; (b) the summarized receiver operating characteristic curves.

**Figure 6:**
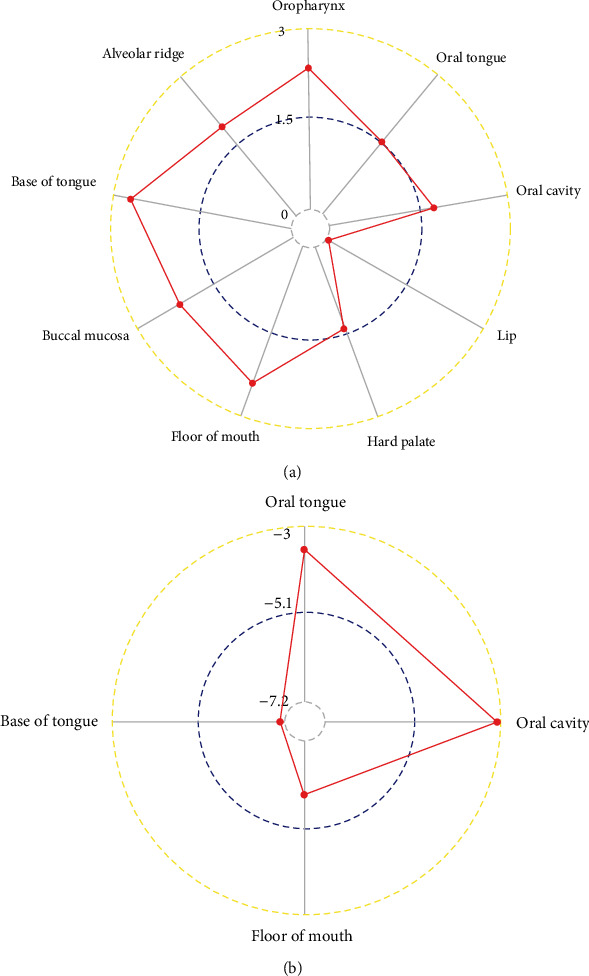
Distribution of ZIC2 expression in oral cancer samples of different parts and various noncancer oral tissues: (a) radar plot for ZIC2 expression in various oral cancer samples; (b) radar plot for ZIC2 expression in noncancer oral tissues.

**Figure 7:**
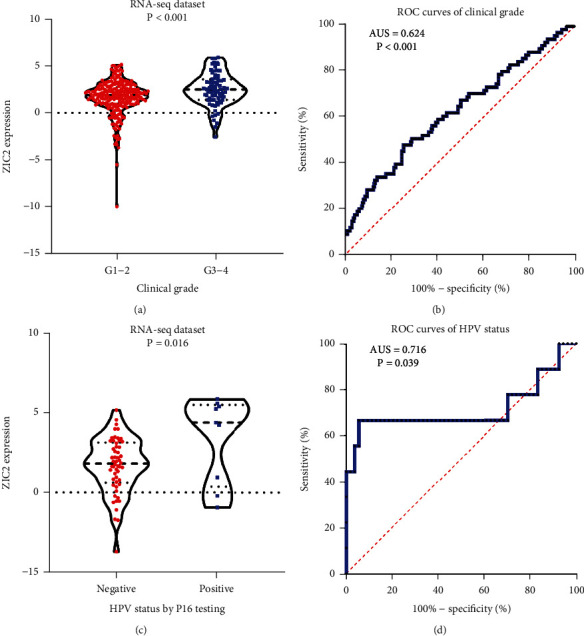
Significant relationships between ZIC2 expression and the clinicopathological variables of oral cancer patients: (a) box plot for ZIC2 expression value in oral cancer patients of high or low clinical grade; (b) ROC curves of the discerning ability of ZIC2 expression for oral cancer patients with high clinical grade; (c) box plot for ZIC2 expression value in oral cancer patients of positive or negative HPV status; (d) ROC curves of the discerning ability of ZIC2 expression for oral cancer patients with positive HPV status.

According to the statistical analysis for expression data of in-house tissue microarray, ZIC2 expression was significantly higher in 159 oral cancer tissues compared with that in 48 noncancer oral tissues (7.89 ± 1.994; 1.46 ± 1.868, *P* < 0.05) (Figure 1(g)). The upregulation of ZIC2 demonstrated preferable discriminating ability for oral cancer tissues (AUC = 0.980) (Figure 1(h)). Specifically, oral cancer patients with lymph node metastasis bore obviously higher ZIC2 expression compared with oral cancer patients without lymph node metastasis (*P* < 0.001) (Figure 1(i). The aberrant expression of ZIC2 in oral cancer is capable of differentiating between oral cancer patients with or without lymph node metastasis (Figure 1(j)). In addition to in-house tissue microarray, a total of 22 microarrays in GEO database and RNA-seq dataset from TCGA were enrolled for compilation of expression profiles (Figure 2) (Table 1). Multiple microarrays from the same GPL platforms such as GPL14951, GPL5175, and GPL6480 were merged as a whole. Therefore, 17 expression matrixes were prepared for calculation of SMD values. The landscape of ZIC2 expression and the distinguishing ability of it for oral cancer in the 17 expression matrixes is displayed in Figures 3 and 4. The integrated expression analysis results for all in-house and external datasets confirmed overexpression of ZIC2 in oral cancer as well as the good distinguishing performance (SMD = 1.31, 95% CI = 0.73 − 1.89; AUC = 0.89) (Figure 5). The expression pattern of ZIC2 in oral cancer and noncancer oral tissues of different parts from RNA-seq dataset was also analyzed. We found high level of ZIC2 expression oral cancers on the base of the tongue, buccal mucosa, oropharynx, and normal oral cavity and oral tongue tissues. Relatively lower ZIC2 expression was observed in oral cancers of oral cavity, lip, hard palate, and normal base of the tongue and floor of mouth tissues (Figure 6). Association analysis of ZIC2 expression and clinicopathological parameters of oral cancer patients revealed remarkable correlation between ZIC2 expression and higher clinical grade and positive HPV status (*P* < 0.05) (Figure 7).

### 3.2. The Prognostic Stratification Ability of ZIC2 Expression for Oral Cancer Patients

**Figure 8:**
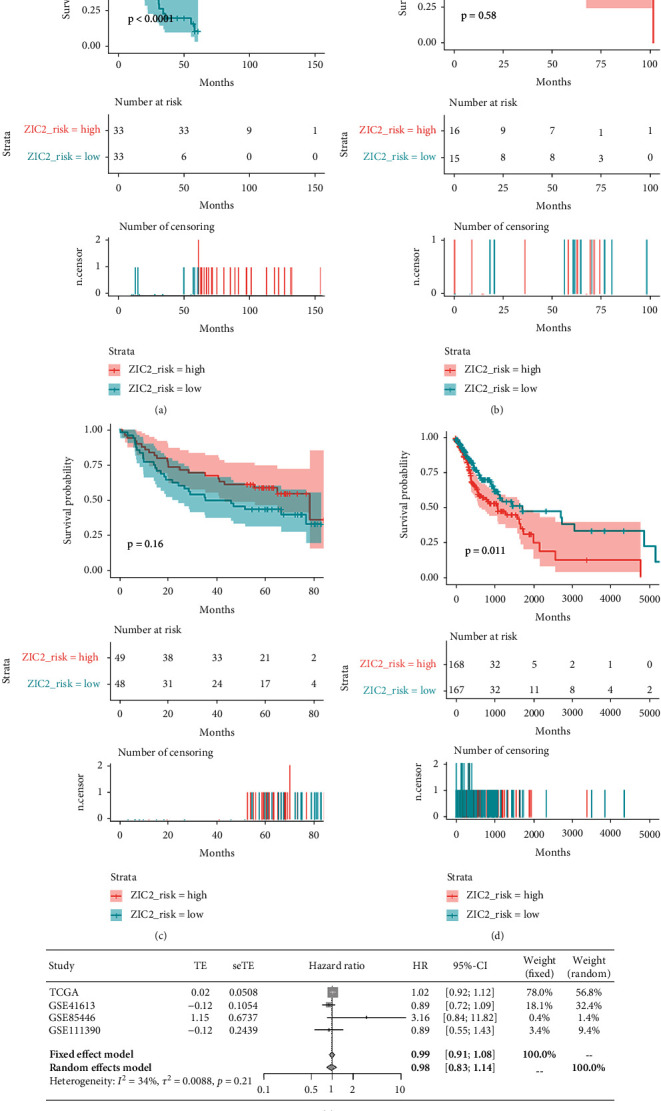
The prognostic value of ZIC2 expression for oral cancer patients. Kaplan-Meier survival curves of oral cancer patients with high or low ZIC2 expression from GSE85446 (a), GSE111390 (b), GSE41613 (c), and TCGA database (d); (e) forest plot of hazard ratio values yielded from univariate Cox regression analysis.

The prognostic significance of ZIC2 expression was analyzed in four datasets including RNA-seq dataset in TCGA database, GSE41613, GSE85446, and GSE111390. While oral cancer patients with lower expression of ZIC2 have improved survival probability than oral cancer patients with higher expression of ZIC2 in TCGA dataset (*P* = 0.011), the overexpression of ZIC2 in GSE85446 indicated worse overall survival of oral cancer patients (*P* < 0.001) (Figures 8(a)–8(d)). Neither the univariate Cox regression analysis for individual dataset nor the summarization for all HR values reported significant results (Figure 8(e)).

### 3.3. The Subcellular Localization and Coexpressed Genes of ZIC2

**Figure 9:**
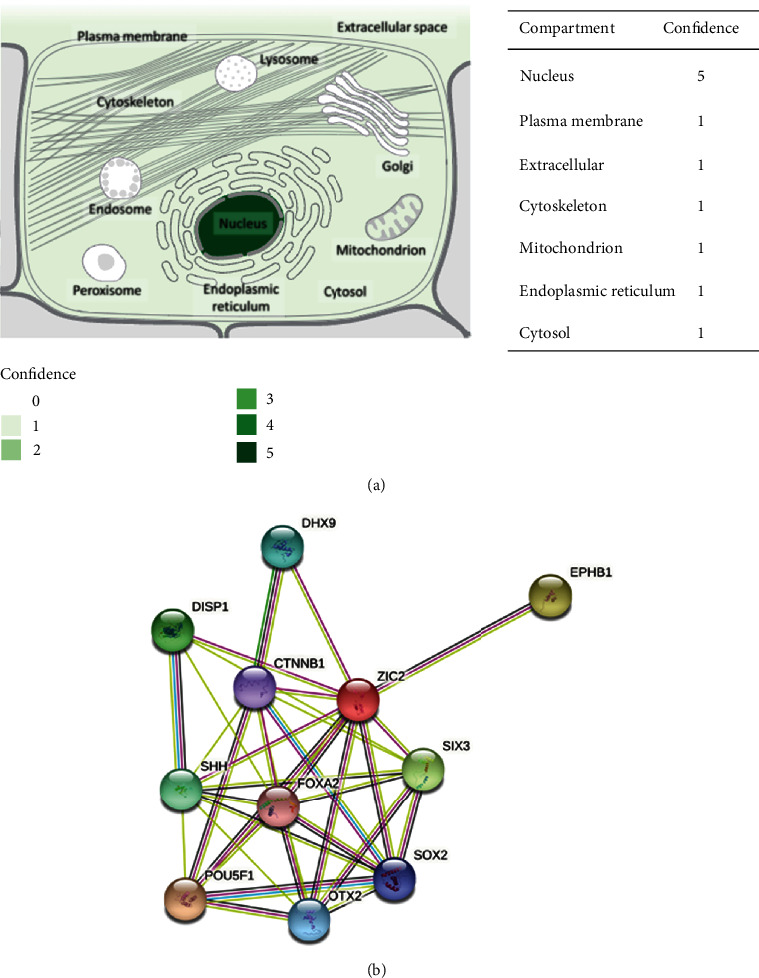
Localization of ZIC2 in eukaryotic cells and functional partners of ZIC2 in oral cancer: (a) distribution diagram of ZIC2 expression abundance in eukaryotic cells; (b) interaction network of ZIC2 and its functional partners.

ZIC2 gene was mostly located in nucleus and sparsely appeared in other compartments such as plasma membrane, cytoskeleton, mitochondrion, and endoplasmic reticulum (Figure 9(a)). Ten genes including DHX9, EPHB1, SIX3, SOX2, OTX2, POU5F1, SHH, FOXA2, CTNNB1, and DISP1 were predicted as the functional partners of ZIC2 (Figure 9(b)).

### 3.4. The Upstream Regulators and Downstream Transcriptional Binding Sites of ZIC2

**Figure 10:**
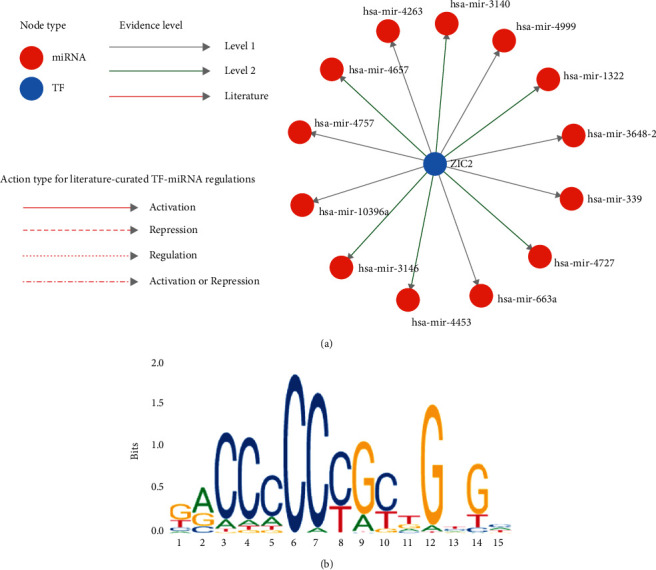
Upstream and downstream transcriptional regulation mechanism of ZIC2: (a) miRNA-ZIC2 network; (b) sequence logo of the possible transcription binding sites of ZIC2.

ZIC2 expression might be modulated by multiple miRNAs including miR-3140, miR-4999, miR-1322, and miR-4263 (Figure 10(a)). A total of 12 transcription factor binding sites of ZIC2 with relative scores of >0.8 were predicted by JASPAR database. The sequence logo and other detailed information are listed in Table 2 and Figure 10(b).

### 3.5. The Associations between ZIC2 Expression and Immune Infiltration of Oral Cancer

**Figure 11:**
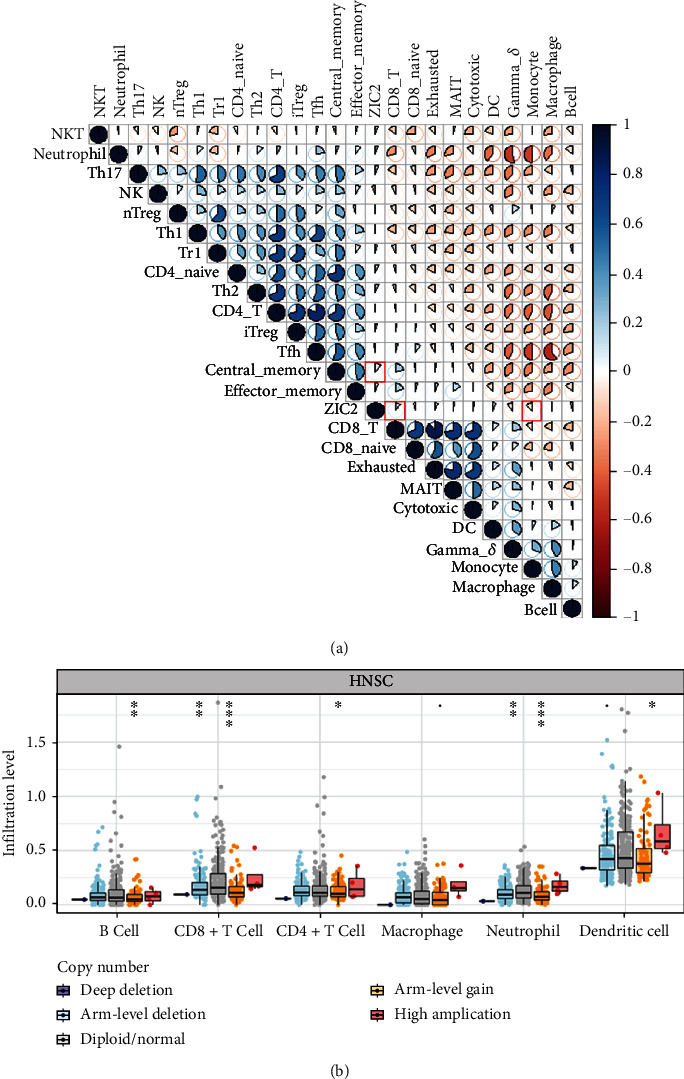
Correlation between ZIC2 expression and immune infiltration of oral cancer or head and neck cancer: (a) correlation plot of the associations between ZIC2 expression and the infiltration level of 24 immune cells calculated through ImmunecellAI. Significant correlation pairs were marked in red; (b) box plot displaying immune infiltration level of six immune cells in head and neck cancer groups with different copy numbers of ZIC2. ^∗^ : *p* < 0.05; ^∗∗^ : *p* < 0.01; ^∗∗∗^ : *p* < 0.001.

Correlation analysis for ZIC2 expression and 24 immune cells indicated significant positive associations between ZIC2 expression and the infiltration level of CD8+ T and central memory cells (*r* = 0.119, *P* = 0.028; *r* = 0.107, *P* = 0.048) as well as the negative associations between expression and the infiltration level of monocytes (*r* = −0.121, *P* = 0.026) (Figure 11(a)). With regard to immune infiltration in oral samples with different copy numbers of ZIC2, the infiltration level of CD8+ T cell, dendritic cells, and neutrophil was notably higher in oral cancer samples with high amplification than that in other types of oral cancer (*P* < 0.05). The infiltration level of six immune cells in oral cancer samples with arm-level deletion, diploid/normal, or arm-level gain showed no significant difference. Less infiltration level of six immune cells was detected in oral cancer samples with deep deletion (Figure 11(b)).

### 3.6. Drug Sensitivity Prediction for Oral Cancer Patients with Different Levels of ZIC2 Expression

**Figure 12:**
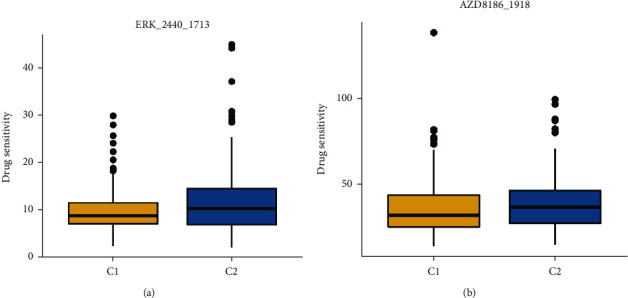
Drug sensitivity of oral cancer patients with high or low expression of ZIC2 to two compounds: (a) box plot of sensitivity scores predicted for ERK_2240; box plot of sensitivity scores predicted for AZD8186.

It was predicted that oral cancer patients with relatively higher ZIC2 expression were more sensitive to two compounds including AZD8186 and ERK_2240 than oral cancer patients with lower ZIC2 expression (*P* < 0.05) (Figure 12). The scores of drug sensitivity for each oral cancer sample to 179 compounds are recorded in Supplementary Table 2.

### 3.7. Functional Annotations for Genes Differentially Expressed in High and Low ZIC2 Expression Groups

**Figure 13:**
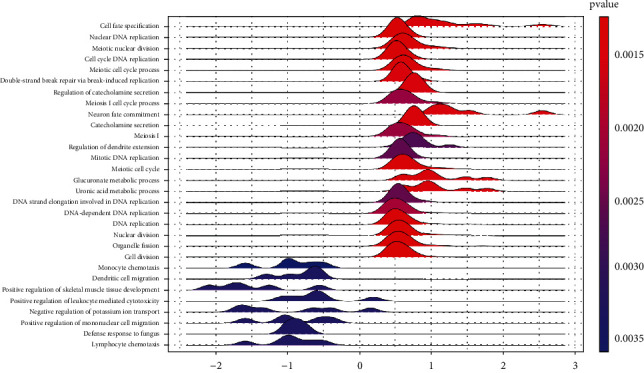
Ridge plot for significantly enriched gene ontology terms. The *x*-axis reflected the enrichment scores of these terms.

A total of 9764 genes with available Entrez IDs presented significant differential expression between high and low ZIC2 expression groups (Supplementary Table 3). Biological processes and molecular functions such as cell fate specification, nuclear DNA replication, and meiotic nuclear division were significantly enriched by genes upregulated in the high ZIC2 expression group, while biological processes and molecular functions including cell division, monocyte chemotaxis, and dendritic cell migration were significantly enriched by genes downregulated in the low ZIC2 expression group (Figure 13). No KEGG pathway terms were significantly clustered by the differentially expressed genes.

### 3.8. The Influence of Knocking Down ZIC2 Expression on the Cellular Functions of Cal-27 Cells

**Figure 14:**
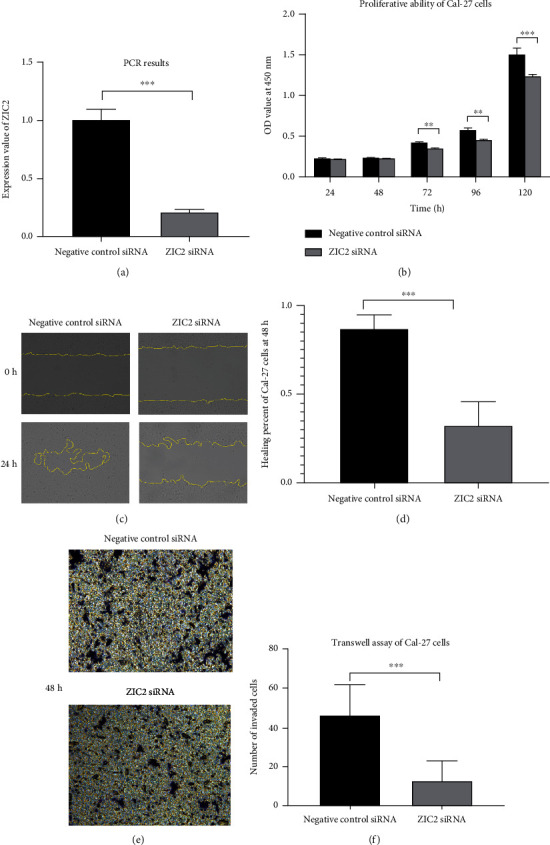
The effect of knocking down ZIC2 expression on biological functions of Cal-27 cells: (a) expression of ZIC2 mRNA between Cal-27 cells transfected with negative control (NC) RNA and ZIC2 siRNA; (b) box plot of OD values from CCK8 assay; (c) images of cell migration around scratches; (d) box plot of wound healing percentage at 48 h; (e) images of cells passing through the basement membrane of Transwell chambers; (f) box plot of number of invaded cells at 48 h. ^∗∗^*P* < 0.01; ^∗∗∗^*P* < 0.001.

The results from in vitro experiments suggested that transfection of ZIC2 siRNA successfully reduced the mRNA expression of ZIC2 and knocking down of ZIC2 expression significantly impacted the proliferation rate of Cal-27 cells, especially at 72 and 96 h (*P* < 0.05, Figures 14(a) and 14(b)). The number of migrated Cal-27 cells and number of cells passing through the basement membrane of Transwell chambers also remarkably reduced in the group of transfection with ZIC2 siRNA compared with the control group (*P* < 0.05) (Figures 14(c)–14(f)).

## 4. Discussion

The molecular regulatory network of oral cancer was far from elucidated, which limits the improvement of clinical treatment. More effective biomarkers with clinical importance for oral cancer await to be discovered. Herein, the anticancer potential of ZIC2 in a wide type of human cancers inspired us that ZIC2 might also showed abnormal expression in oral cancer and played crucial roles in the occurrence and progression of oral cancer.

In the current study, the expression characteristics and clinicopathological value of ZIC2 were first analyzed through in-house tissue microarray and further confirmed by large samples of global microarrays and RNA-seq datasets. Expression data from the above sources unanimously proved the significant overexpression of ZIC2 in oral cancer. Particularly, ZIC2 protein showed mainly cytoplasmic staining in the current work. Although most transcription factors are localized mainly in nucleus, a few of transcription factors were found to localize in the cytoplasm. The study of Le Magnen et al. reported the cytoplasmic location of transcription factor Klf4 with in prostate cancer, and it was related to an oncogenic differentially spliced isoforms of Klf4 without nuclear localization signal encoded in exon three [19], which might also explain the mainly cytoplasmic staining of ZIC2 in the present study. As for the dark staining seen in the basal cell layer of normal oral epithelium, because basal cells are in a state of active proliferation and have strong regeneration ability, immunohistochemical staining of ZIC2 protein might also be observed in the basal cell layer of normal oral epithelium, which was weaker than that in oral cancer cells. The relatively higher expression level of ZIC2 presented by lymph node metastasis group, advanced clinical grade group, and positive HPV status group implicated that ZIC2 overexpression might promote the clinical progression of oral cancer. It could be preliminarily inferred from the differential expression and clinicopathological significance analysis that ZIC2 may act as an oncogenic factor in the tumorigenesis of oral cancer. To test the performance of overexpressed ZIC2 in risk stratification of the overall survival probability of oral cancer patients, we carried out survival analysis and the results revealed no significant prognostic value of ZIC2 expression for oral cancer patients. Thus, the association between ZIC2 expression and the prognosis of oral cancer patients could not be firmly established based on the analysis results in the present study.

For better understanding of the molecular basis of the oncogenic potential of ZIC2 overexpression in oral cancer, we performed a series of multifaceted analysis expanding from ZIC2, to the coexpressed genes, transcriptional regulators, immune infiltration, and genes correlated with ZIC2 in oral cancer. The chief distribution of ZIC2 in cell nucleus corresponded with the function of regulating the spatiotemporal specific expression of target genes by ZIC2 as a transcription factor [20]. Coexpressed genes of ZIC2 calculated from STRING database revealed the interactions between ZIC2 and its functional partners. It is worth noting that several of the functional partners including CTNNB1, SHH, FOXA2, and SOX2 have been reported to be involved in the progression of oral cancer or linked with the stem cell phenotype of oral cancer cells [21–24]. Hereby, we hypothesized that ZIC2 might also coact with these functional partners to affect the biological behaviors of oral cancer cells. We also identified miRNAs that might modulate the expression of ZIC2 and the theoretical transcriptional binding site of ZIC2, which implied potential regulation axis of transcription and expression centered around ZIC2 in oral cancer. The tumor microenvironment (TME) encompasses cancer cells, extracellular stroma, and noncancerous cells and exerts critical influence on biological functions and chemoresistance of cancer cells [25]. Immunotherapy directed at TME has been proven to be an effective therapeutic option in treating recurrent or metastatic head and neck cancer [26]. Therefore, we evaluated the relationships between ZIC2 expression or copy number and the infiltration of immune cells in oral cancer or head and neck cancer. The significant higher infiltration level of five types of immune cells in oral cancer samples with high amplification of ZIC2 and positive links between ZIC2 expression and the infiltration levels of CD8+ T cells as well as central memory cells indicated that overexpression of ZIC2 in oral cancer samples might stimulate the antitumor immunity responses. After obtaining the analysis results of the oncogenic potential of ZIC2 and its remarkable correlation with immune infiltration in oral cancer, we were curious about drugs which might show improved efficacy in oral cancer patients with high expression of ZIC2 and scored the drug sensitivity. With respect to the two compounds that were predicted to present higher affinity to oral cancer patients with higher ZIC2 expression, AZD8186 is a selective phosphoinositide 3-kinase/AKT/mTOR inhibitors that demonstrated antitumor potency in various PTEN-deficient tumors [27]; there is no documented pharmacological effect of ERK_2440 in human cancers. It would be interesting to investigate the impact of the two compounds on the expression level and oncogenic effect of ZIC2 in oral cancer in future studies. Finally, we concentrated on genes differentially expressed between low and high ZIC2 expression groups of oral cancer and explored their functional enrichment in oral cancer. The recorded list of significant terms reflected the active roles of ZIC2 in cell fate specification, nuclear DNA replication, and cell division and meiotic cell cycle process. The abnormality of the above molecular functions and biological processes was all closely related to the oncogenesis of oral cancer [28–31]. From which, we speculated that the oncogenic roles of ZIC2 in oral cancer might be intimately linked with the involvement of ZIC2 in the above molecular functions, biological process, and KEGG pathways. The results from in vitro experiments in the present work verified the essential role of ZIC2 in the proliferation, migration, and invasion of oral cancer; nevertheless, more experiments were needed for exploring the function of ZIC2 in other biological processes and KEGG pathways of oral cancer.

Another limitation of the current work was the inclusion of samples from different regions of the head and neck cancer. The key research objects of this study were oral cancer samples in a narrow sense; however, oropharyngeal and hypopharyngeal cancer samples were also included for supplement of sample size. The accumulation of oral cancer samples from the tongue, floor of the mouth, palate, gingiva, cheek, and alveolar mucosa was needed for more targeted research findings.

In conclusion, the holistic analysis in the present work suggested the promoting effect of ZIC2 on the clinical advancement of oral cancer and the significant correlations between ZIC2 upregulation and the immune infiltration of oral cancer, which pointed to the clinical value of ZIC2 in oral cancer.

## Figures and Tables

**Table 1:** Detailed information of all included microarrays and RNA-seq dataset in the current study.

ID	Included gene chips	Year	Number of samples		Experiment type	Contact author
			Oral cancer tissues	Noncancer oral tissues		
GPL14951	GSE34105	2012	89	31	Expression profiling by array	Matilda Rentoft
	GSE34106	2012			Expression profiling by array	Matilda Rentoft

GPL5175	GSE25099	2011	100	22	Expression profiling by array	Chien-Hua Peng
	GSE41116	2013			Expression profiling by array	Jianjun Zhang

GPL6480	GSE84846	2017	221	34	Expression profiling by array	Nicoletta Bertani
	GSE85446	2017			Expression profiling by array	Daoud Sie
	GSE23558	2011			Expression profiling by array	Manoj Balkrishna Mahimkar
	GSE142583	2021			Expression profiling by array	Paola Ostano

GPL8300	GSE13601	2008	31	26	Expression profiling by array	Nicholas D. Socci

GPL6947	GSE19089	2009	3	3	Expression profiling by array	Yan W. Asmann

GPL10526	GSE31056	2011	23	73	Expression profiling by array	Levi Waldron

GPL2986	GSE36090	2012	13	3	Expression profiling by array	Koh-ichi Nakashiro

GPL6883	GSE37991	2013	40	40	Expression profiling by array	Chia Huei Lee

GPL8490	GSE46802	2013	6	10	Expression profiling by array	Rebecca M. Towle

GPL10739	GSE56532	2014	10	6	Expression profiling by array	Sivapriya Pavuluri

GPL18281	GSE75538	2016	14	14	Expression profiling by array	Binay Panda

GPL18282	GSE75539	2016	7	8	Expression profiling by array	Binay Panda

GPL17077	GSE146483	2020	8	3	Expression profiling by array	Yutaro Kase

GPL24676	GSE193205	2022	3	3	Expression profiling by high-throughput sequencing	Stefano Scalera

GPL24676	GSE184616	2021	15	15	Expression profiling by high-throughput sequencing	Dario Strbenac

GPL18180	GSE160042	2021	10	10	Expression profiling by array	Zehang Zhuang

TCGA	NA	NA	341	32	RNA-sequencing	—

**Table 2:** The predicted transcription factor binding sites for ZIC2.

Matrix ID	Name	Score	Relative score	Sequence ID	Start	End	Strand	Predicted sequence
MA0751.1	MA0751.1.ZIC4	16.149668	0.934287566	NC_000013.11:99979783-99981783	230	244	-	CGCCCCCCGCGGCGT
MA0751.1	MA0751.1.ZIC4	12.8101	0.888787631	NC_000013.11:99979783-99981783	402	416	+	AGCCCCCCGCCGCTC
MA0751.1	MA0751.1.ZIC4	10.017402	0.850738528	NC_000013.11:99979783-99981783	187	201	-	CCCCACCCGCCGCTT
MA0751.1	MA0751.1.ZIC4	9.545151	0.844304347	NC_000013.11:99979783-99981783	1629	1643	+	GCGCCCCCGCCGCGG
MA0751.1	MA0751.1.ZIC4	9.534228	0.844155534	NC_000013.11:99979783-99981783	1235	1249	-	TCCCTCCCGCCGGTC
MA0751.1	MA0751.1.ZIC4	8.675149	0.83245101	NC_000013.11:99979783-99981783	758	772	-	GCCATCCTGCCGCGC
MA0751.1	MA0751.1.ZIC4	8.247261	0.826621253	NC_000013.11:99979783-99981783	707	721	-	GGCCGACCGCGGCGC
MA0751.1	MA0751.1.ZIC4	7.714408	0.819361397	NC_000013.11:99979783-99981783	1558	1572	-	CCTCCCCCGCGGCCC
MA0751.1	MA0751.1.ZIC4	7.348327	0.814373731	NC_000013.11:99979783-99981783	848	862	-	GCCCCGCTGCCGGGC
MA0751.1	MA0751.1.ZIC4	7.1283464	0.811376605	NC_000013.11:99979783-99981783	851	865	-	CCCGCCCCGCTGCCG
MA0751.1	MA0751.1.ZIC4	6.4369054	0.801956068	NC_000013.11:99979783-99981783	210	224	-	TCCGCCCCTCGGCGC

## Data Availability

The data in the current study was included in all figures, tables, and supplementary materials.

## Abbreviations

ZIC2: Zic family member 2
IHC: Immunohistochemistry
FPKM: Fragments per kilobase per million
TCGA: The Cancer Genome Atlas
GEO: Gene Expression Omnibus
SMD: Standard mean difference
SROC: Summarized receiver operating characteristics
CI: Confidence interval
HR: Hazard ratio
GDSC: Genomics of Drug Sensitivity in Cancer
GSEA: Gene set enrichment analysis
NES: Normalized enrichment score
AUC: Area under curve.

## Supplementary Materials

Supplementary 1 Figure 1: positive and negative controls used for evaluating IHC staining. (A) Immunohistochemistry pictures of positive control of ZIC2 expression in oral cancer tissues (×100). (B) Immunohistochemistry pictures of positive control of ZIC2 expression in oral cancer tissues (×200). (C) Immunohistochemistry pictures of positive control of ZIC2 expression in oral cancer tissues (×400). (D) Immunohistochemistry pictures of negative control of ZIC2 expression in noncancer oral tissues (×100). (E) Immunohistochemistry pictures of negative control of ZIC2 expression in noncancer oral tissues (×200). (F) Immunohistochemistry pictures of negative control of ZIC2 expression in noncancer oral tissues (×400).

Supplementary 2 Table 1: detailed clinicopathological information of oral cancer patients included for in-house tissue microarray.

Supplementary 3 Table 2: the predicted drug sensitivity of oral samples to 179 compounds.

Supplementary 4 Table 3: differentially expressed between high and low ZIC2 expression groups in RNA-seq dataset.
