# Supplementary figures and images for: Identification of ZIC2 as a Potential Biomarker Linked with the Clinical Progression and Immune Infiltration of Oral Cancer: A Multicenter Study
Source: Int J Genomics. 2024 Jan 24;2024:3256694. doi: 10.1155/2024/3256694 (PMC10830925; doi:10.1155/2024/3256694)

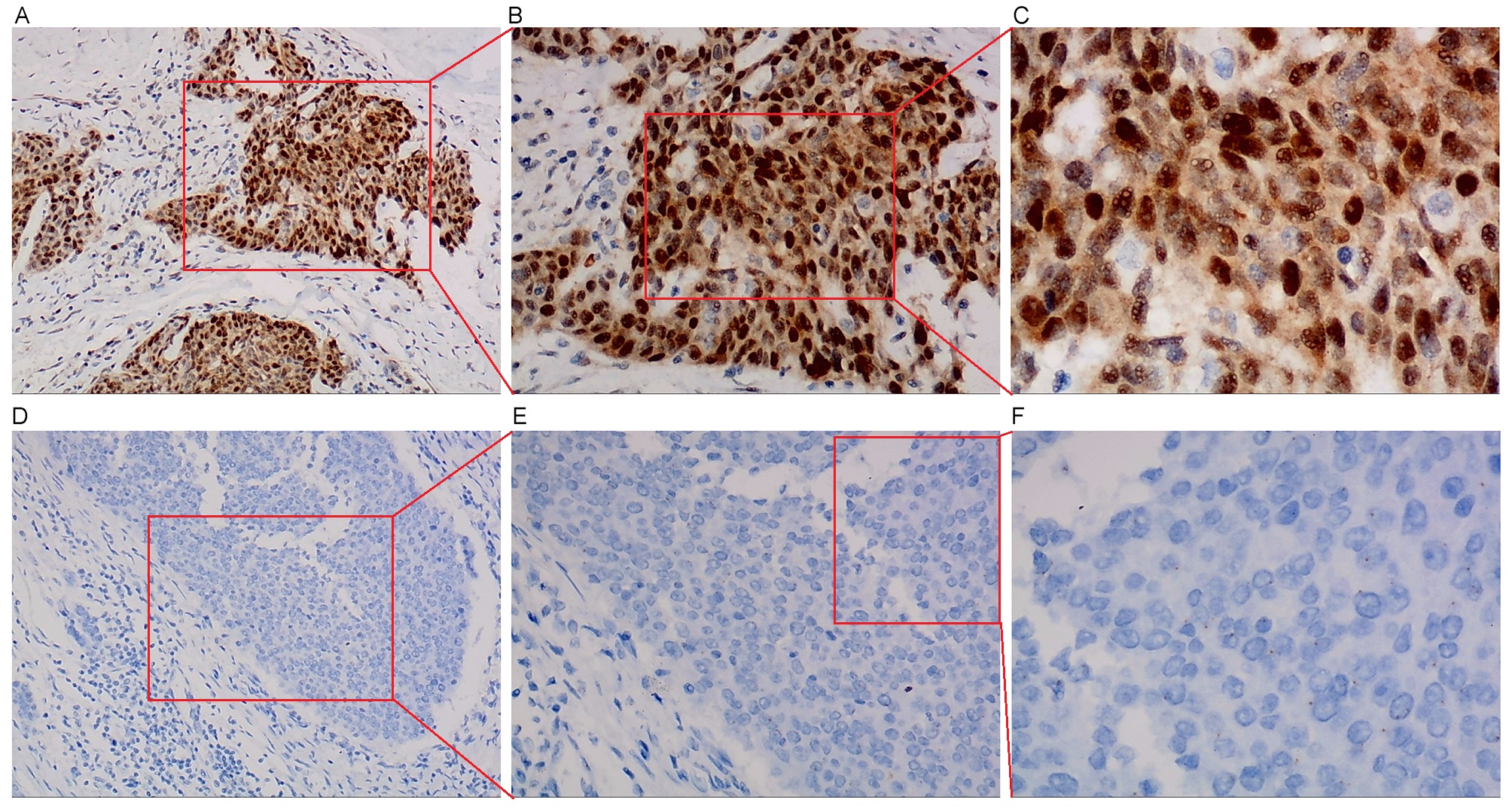

Supplement: Supplementary 1 — Figure 1: positive and negative controls used for evaluating IHC staining. (A) Immunohistochemistry pictures of positive control of ZIC2 expression in oral cancer tissues (×100). (B) Immunohistochemistry pictures of positive control of ZIC2 expression in oral cancer tissues (×200). (C) Immunohistochemistry pictures of positive control of ZIC2 expression in oral cancer tissues (×400). (D) Immunohistochemistry pictures of negative control of ZIC2 expression in noncancer oral tissues (×100). (E) Immunohistochemistry pictures of negative control of ZIC2 expression in noncancer oral tissues (×200). (F) Immunohistochemistry pictures of negative control of ZIC2 expression in noncancer oral tissues (×400). [file 3256694.f1.jpg]
